# Aortic aneurysms and markers of platelet activation, hemostasis, and endothelial disruption in people living with HIV

**DOI:** 10.3389/fimmu.2023.1115894

**Published:** 2023-02-02

**Authors:** Sylvester Klöcker Grønbæk, Julie Høgh, Andreas Dehlbæk Knudsen, Michael Huy Cuong Pham, Per Ejlstrup Sigvardsen, Andreas Fuchs, Jørgen Tobias Kühl, Lars Køber, Jan Gerstoft, Thomas Benfield, Sisse Rye Ostrowski, Klaus Fuglsang Kofoed, Susanne Dam Nielsen

**Affiliations:** ^1^ Department of Infectious Diseases, Copenhagen University Hospital, Rigshospitalet, Copenhagen, Denmark; ^2^ Department of Cardiology, The Heart Center, Copenhagen University Hospital, Rigshospitalet, Copenhagen, Denmark; ^3^ Department of Clinical Medicine, University of Copenhagen, Copenhagen, Denmark; ^4^ Center of Research and Disruption of Infectious Diseases, Department of Infectious Diseases, Copenhagen University Hospital, Amager and Hvidovre, Hvidovre, Denmark; ^5^ Department of Clinical Immunology, Copenhagen University Hospital, Rigshospitalet, Copenhagen, Denmark; ^6^ Department of Radiology, Copenhagen University Hospital, Rigshospitalet, Copenhagen, Denmark

**Keywords:** HIV, PLWH, aortic aneurysm, sCD40L, D-dimer (DD), syndecan-1, thrombomodulin (TM)

## Abstract

**Introduction:**

People living with HIV (PLWH) are at twice the risk of developing cardiovascular diseases and have more than four times higher odds of aortic aneurysm (AA) than the uninfected population. However, biomarkers of AA in PLWH are yet to be discovered. We aimed to investigate whether circulating biomarkers reflecting platelet activation, hemostasis and endothelial disruption, i.e. sCD40L, D-dimer, syndecan-1, and thrombomodulin, were associated with AA in PLWH.

**Methods:**

Five hundred seventy one PLWH from the Copenhagen Comorbidity in HIV Infection (COCOMO) study ≥40 years of age with an available contrast-enhanced CT scan as well as available biomarker analyses were included. The biomarkers were analyzed on thawed plasma. For each biomarker, we defined high level as a concentration in the upper quartile and low level as a concentration below the upper quartile. For D-dimer, the cut-off was defined as the lower limit of detection. Using unadjusted and adjusted logistic and linear regression models, we analyzed associations between AA and sCD40L, D-dimer, syndecan-1, and thrombomodulin, respectively in PLWH.

**Results:**

PLWH had median (IQR) age 52 years (47-60), 88% were male, median (IQR) time since HIV diagnosis was 15 years (8-23), and 565 (99%) were currently on antiretroviral treatment. High level of sCD40L was associated with lower odds of AA in both unadjusted (odds ratio, OR, 0.23 (95% CI 0.07-0.77; *P*=0.017)) and adjusted models (adjusted OR, aOR, 0.23 (95% CI 0.07-0.78; *P*=0.019)). Detectable level of D-dimer was associated with higher odds of AA in both unadjusted (OR 2.76 (95% CI 1.34-5.67; *P*=0.006)) and adjusted models (aOR 2.22 (95% CI 1.02-4.85; *P*=0.045)).

**Conclusions:**

SCD40L was associated with lower odds of AA whereas D-dimer was independently associated with higher odds of AA in PLWH. This calls for further investigations into specific biomarkers to aid early diagnosis of AA in PLWH.

## 1 Introduction

As the median age of people living with HIV (PLWH) increases (1), so does the burden of age-related comorbidities (2). The risk of incident cardiovascular diseases (CVD) in PLWH is twice that of uninfected individuals (3), with myocardial infarction, stroke, and coronary artery disease being the most frequent manifestations (4). In a previous study by our group, HIV infection was associated with a 4.5 increased odds ratio (OR) of having aortic aneurysms (AA) compared to uninfected controls (5). AA is a rare but potentially severe manifestation of CVD, as the mortality rate of a ruptured abdominal AA may be as high as 80% (6). The European Society of Cardiology recommends screening of populations with a high risk of abdominal AA (7), because most AA are asymptomatic and diagnosed incidentally (8).

The initial phase of the pathogenesis of AA is not fully understood, but studies have suggested endothelial cell injury to contribute (9, 10). Associations have been found between AA, in the majority of studies in patients undergoing surgery for AA, and a marker of platelet activation, soluble CD40L (sCD40L) (11, 12), a marker of hemostasis, D-dimer (13–17), and the markers of endothelial disruption, syndecan-1 (18–20) and soluble thrombomodulin (21, 22), respectively. Importantly, all of these biomarkers have been reported to be elevated in PLWH compared to uninfected controls (23–28).

Possible associations between these biomarkers and AA have yet to be established but could aid in the early identification of high-risk patients. So far, no studies on the association between AA and markers of platelet activation, hemostasis, and endothelial disruption, respectively, in PLWH have been published. Thus, we investigated whether sCD40L, D-dimer, syndecan-1, and thrombomodulin (the biomarkers) are associated with AA in a large cohort of well-treated PLWH. We hypothesized that each of the biomarkers would be independently associated with AA in PLWH.

## 2 Materials and methods

### 2.1 Study design and population

The Copenhagen Comorbidity in HIV Infection (COCOMO) study is a non-interventional cohort study that aims to assess non-AIDS comorbidities in PLWH. Inclusion criteria were age above 18 years and a positive HIV test. Between March 2015 and December 2016, 1099 PLWH living in the greater Copenhagen area were included. Procedures for recruitment and collection of data have previously been described elsewhere (29). A combined thoracic and abdominal contrast-enhanced computed tomography (CT) scan was offered to all COCOMO participants. In this study, we included COCOMO participants who were ≥40 years of age, had a contrast-enhanced CT performed, and had biomarkers measured in plasma.

Written informed consent was obtained from all participants. Ethical approval was obtained by the Regional Ethical Committee of Copenhagen (COCOMO: H-8-2014-004). The study was carried out in accordance with the Declaration of Helsinki.

### 2.2 Clinical characteristics and self-reported outcome

A physical examination including measurement of blood pressure and anthropometrics was performed by trained clinical staff. Venous blood was collected non-fasting. Blood for plasma samples (EDTA anti-coagulated) was stored on ice until centrifugation in a cold centrifuge at 4˚C, and cryovials were transferred to liquid nitrogen within 72 hours (29). Extensive questionnaires included questions regarding medication, medical history, and smoking. From medical records, HIV-specific data such as transmission mode, duration of HIV infection, type of antiretroviral therapy, and hepatitis B and C status were obtained. Hepatitis B virus co-infection was defined as positive hepatitis B virus surface-antigen and hepatitis C virus co-infection as positive hepatitis C virus RNA. Hypertension was defined as systolic blood pressure ≥140 mmHg and/or diastolic blood pressure ≥90 mmHg and/or current use of antihypertensive medication according to Joint National Committee guidelines (30).

### 2.3 CT examinations and aortic analyses

Contrast-enhanced thoracic and abdominal CT examinations including contrast-enhanced CT angiography were performed using a 320-detector CT scanner (Aquilion ONE, ViSION Edition, Canon Medical Systems, Otawara, Japan) at Rigshospitalet University Hospital, Copenhagen, Denmark.

Aortic analyses were performed by two trained examiners on contrast-enhanced CT images. Maximal and minimal inner aortic diameter was measured at seven anatomical points of the aorta, in four of which maximal outer diameter of the aorta was measured as well (5). According to the European Society of Cardiology guidelines, aortic aneurysms were defined as ≥50% increase in aortic diameter compared to the expected normal diameter or an infrarenal diameter of ≥30 mm (7). This resulted in the definition of AA as diameter of ascending aorta ≥45 mm and/or diameter of descending aorta ≥35 mm and/or diameter of suprarenal aorta ≥30 mm and/or diameter of the infrarenal aorta ≥30 mm (31).

### 2.4 Markers of platelet activation, hemostasis, and endothelial disruption

Plasma concentrations of sCD40L, soluble syndecan-1, and thrombomodulin, were analyzed on thawed plasma using Luminex^®^ Human Discovery Assays (R&D Systems, UK, Europe) in a 1:2 dilution, according to the manufacturer’s instructions, at the Department of Clinical Immunology, Copenhagen University Hospital, Rigshospitalet, Copenhagen, Denmark. Plasma concentrations of D-dimer were measured as routine biochemistry on fresh blood samples at Department of Clinical Biochemistry, Copenhagen University Hospital, Herlev, Copenhagen, Denmark (29).

### 2.5 Statistics

Continuous data were reported using means and standard deviations for normal deviates and medians with interquartile ranges for variables not normally distributed, as appropriate. For categorical data, frequency counts and percentages of subjects within each category were reported. Logistic regression analyses were applied where the dependent variable was binary and linear regression where the dependent variable was continuous.

As the primary outcome, we investigated the association between AA and high levels of sCD40L, syndecan-1, and thrombomodulin and detectable level of D-dimer one at a time using simple and multivariable logistic regression. For each biomarker, we created a dichotomous variable defining high level of the biomarker as a concentration in the upper quartile and low level as a concentration below the upper quartile. We used the third quartile as cut-off in the dichotomous variables for sCD40L, syndecan-1 and thrombomodulin to make the effect estimates more easily interpretable. For D-dimer, the cut-off was defined as the lower limit of detection (290 ng/mL (Fibrin Equivalent Units, FEU)), creating a dichotomous variable with detectable versus undetectable as most of the measurements were below the lower limit of detection. We created two predefined models; model 1 adjusted for age (per decade) and sex and model 2 adjusted for traditional risk factors that were significant in previous analyses on AA in PLWH (5) (age, sex, BMI category, hypertension, and smoking (current/previous/never)). Furthermore, in sensitivity analyses, we investigated the associations between AA, sCD40L, and (one at a time) use of aspirin, use of statins, weekly alcohol intake, and hypertension. As a sensitivity analysis, we included all four biomarkers into a single analysis with AA.

Additionally, continuous variables were used in pre-specified, secondary analyses to make sure any linear associations between exposure and outcome would not be overlooked. We adjusted for the same predefined models 1 and 2. Moreover, in sensitivity analyses, we investigated associations between aortic diameter (maximum outer diameter of ascending, descending, suprarenal, and infrarenal aorta) and high versus low levels of the biomarkers. Lastly, in sensitivity analyses, we examined associations between aortic wall thickness (maximal outer minus maximal inner diameter of ascending, descending, suprarenal, and infrarenal aorta) and high versus low levels of the biomarkers.

P-values <0.05 were considered statistically significant, and all P-values were two-sided. R (version 4.1.0 (2021-05-18)) was used for all statistical analyses (32).

## 3 Results

We included 571 participants aged 40 years or older with available contrast-enhanced CT and biomarker measurements. As reported previously, 43 aneurysms were found in 39 PLWH (6.8%). The median (IQR) age of PLWH was 52 years (47-60) and 88% were male (**Table 1**). The median (IQR) time since HIV diagnosis was 15 years (8-23) and 565 (99%) were currently on antiretroviral therapy (**Table 1**). Participant characteristics, HIV-specific variables, and biomarkers are listed in **Table 1**.

**Table 1:** Participant characteristics, HIV-specific risk factors, and concentrations of the biomarkers.

Variable	PLWH (N=571)
Characteristics	
Age, median [IQR]	51.6 [47.0-59.8]
Male sex, n (%)	503 (88.1%)
BMI, mean (SD)	24.8 (3.5)
BMI classification, n (%)	
Underweight	14 (2.5%)
Normal weight	301 (52.7%)
Pre-obesity	211 (37.0%)
Obesity	43 (7.5%)
Smoking status, n (%)	
Never smoker	188 (32.9%)
Current smoker	152 (26.6%)
Previous smoker	219 (38.4%)
Hypertension, n (%)	263 (46.1%)
Platelets, **×**10^9^/L, mean (SD)	226.8 (57.5)
HIV-specific risk factors	
Transmission mode, n (%)	
MSM	415 (72.7%)
Heterosexual	116 (20.3%)
IDU	6 (1.1%)
Other	28 (5%)
Current CD4^+^, cells/µL, median [IQR]	680 [520-870]
<200	7 (1.2%)
200-349	34 (6.0%)
350-499	79 (13.8%)
≥500	446 (78.1%)
CD4^+^ nadir **<**200, n (%)	235 (41.2%)
CD4^+^/CD8^+^-ratio, median [IQR]	0.813 [0.578-1.13]
Time since HIV diagnosis, median [IQR]	15.1 [7.74-22.6]
Time on ART, median [IQR]	12.8 [5.94-17.8]
Currently on ART, n (%)	565 (98.9%)
Viral load **>**50	23 (4.0%)
Hepatitis B-virus co-infection, n (%)	18 (3.2%)
Hepatitis C-virus co-infection, n (%)	33 (5.8%)
Concentrations of the biomarkers	
Syndecan-1, pg/mL, median [IQR]	2070 [1801-2413]
Thrombomodulin, pg/mL, median [IQR]	6598 [5541-7823]
sCD40L, pg/mL, median [IQR]	184 [118-243]
D-dimer, ng/mL (FEU), median [IQR]	290 [290-290]

ART, antiretroviral therapy; IDU, injecting drug use; MSM, men who have sex with men; FEU, fibrin equivalent units.

### 3.1 Aortic aneurysms and the association with markers of platelet activation, hemostasis, and endothelial disruption

We found high levels of sCD40L to be associated with lower odds of AA; univariably with OR 0.23 (95% CI 0.07-0.77; *P*=0.017) and adjusted odds ratio, aOR, 0.23 (95% CI 0.07-0.78; *P*=0.019) adjusted in model 2 (**Table 2**). In a sensitivity analysis, further adjustment of model 2 for use of aspirin did not significantly alter the association between high level of sCD40L and AA (aOR 0.25 (95% CI 0.07-0.87); *P*=0.029). In other sensitivity analyses, use of statins, weekly alcohol intake, and hypertension were not associated with AA and sCD40L.

**Table 2:** Associations between AA and high levels of the biomarkers.

Variable	OR (95% CI), P-value	aOR^a^ (95% CI), P-value	aOR^b^ (95% CI), P-value
sCD40L (high vs. low)	0.23 (0.07,0.77), ***P*=0.017**	0.24 (0.07,0.81), ***P*=0.022**	0.23 (0.07,0.78), ***P*=0.019**
D-dimer **(**detectable vs. undetectable)	2.76 (1.34,5.67), ***P*=0.006**	2.22 (1.02,4.85), ***P*=0.045**	2.24 (0.99,5.04), *P*=0.052
Syndecan-1 (high vs. low)	1.55 (0.77,3.10), *P*=0.219	1.27 (0.61,2.64), *P*=0.515	0.89 (0.40,1.98), *P*=0.767
Thrombomodulin (high vs. low)	1.75 (0.88,3.47), *P*=0.109	1.31 (0.63,2.74), *P*=0.466	1.06 (0.48,2.36), *P*=0.877

^a^Model 1 and ^b^model 2.

Bold values: significant associations.

Detectable level of D-dimer was associated with higher odds of AA, both univariably (OR 2.76 (95% CI 1.34-5.67; *P*=0.006)) and adjusted in model 1 (aOR 2.22 (95% CI 1.02-4.85; *P*=0.045)). Adjusting for traditional risk factors in model 2 did not change the parameter estimate, (aOR 2.24 (95% CI 0.99-5.04; *P*=0.052). There were aortic aneurysms among PLWH with undetectable D-dimer.

Syndecan-1 and thrombomodulin were not associated with AA (**Table 2**).

Including all four biomarkers into a single analysis did not significantly alter the associations compared to the individual associations between the biomarkers and AA.

**Figure 1:**
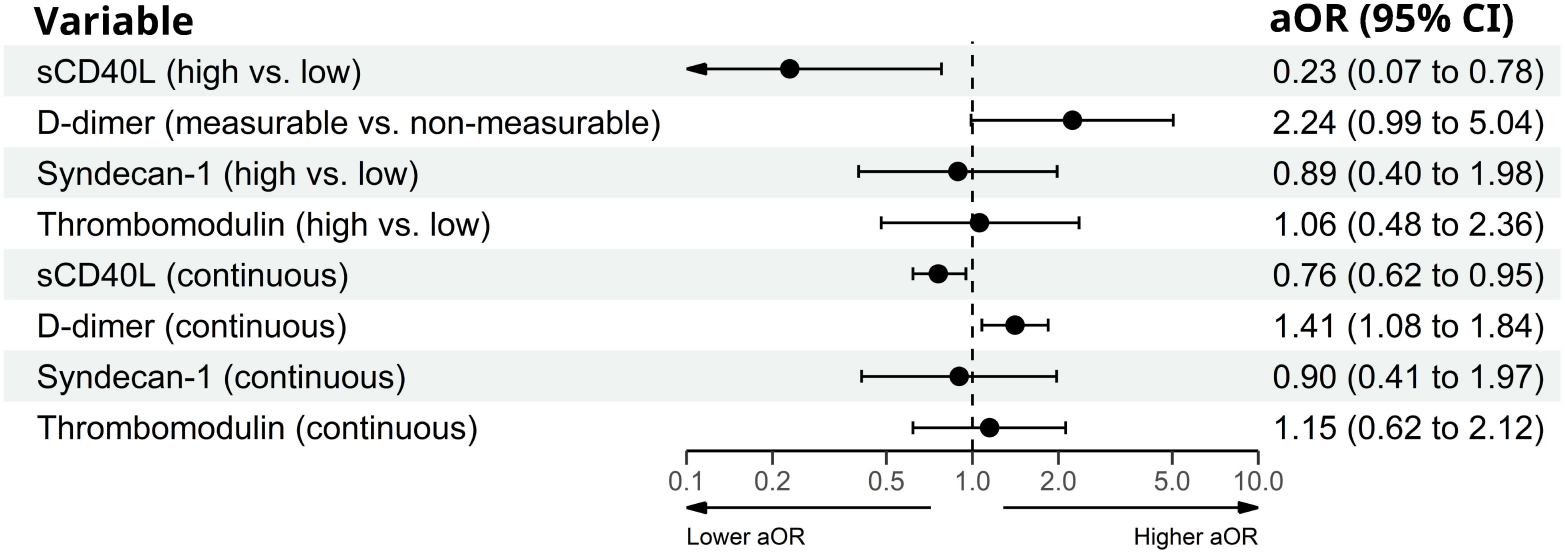
Primary (high vs. low variable) and exploratory (continuous variable, aOR associated with doubling of biomarker) analyses of the association between AA and the biomarkers in PLWH adjusted for age, sex, hypertension, smoking status, and BMI category.

In exploratory analyses with the biomarkers as continuous variables, a doubling in sCD40L concentration was associated with lower odds of AA; univariably with OR 0.81 (95% CI 0.66,0.98; *P*=0.028) and aOR 0.76 (95% CI 0.62-0.95; *P*=0.014) adjusted in model 2. A twofold increase in D-dimer concentration was associated with higher odds of AA; univariably with OR 1.51 (95% CI 1.22-1.86; *P*<0.001) and aOR 1.41 (95% CI 1.08-1.84; *P*=0.010) adjusted in model 2. Syndecan-1 was not significantly associated with AA. A twofold increase in thrombomodulin concentration was univariably associated with higher odds of AA (OR 1.82 (95% CI 1.02-3.26; *P*=0.043) but not in adjusted models (**Table 3**). Analyses of high versus low levels and with continuous variables are shown in **Figure 1**.

**Table 3:** Associations between AA and the biomarkers as continuous variables.

Variable	OR (95% CI), P-value	aOR^a^ (95% CI), P-value	aOR^b^ (95% CI), P-value
sCD40L	0.81 (0.66,0.98), ***P*=0.028**	0.80 (0.65,0.98), ***P*=0.029**	0.76 (0.62,0.95), ***P*=0.014**
D-dimer	1.51 (1.22,1.86), ***P*<0.001**	1.44 (1.13,1.85), ***P*=0.004**	1.41 (1.08,1.84), ***P*=0.010**
Syndecan-1	1.66 (0.85,3.26), *P*=0.139	1.34 (0.66,2.72), *P*=0.417	0.90 (0.41,1.97), *P*=0.787
Thrombomodulin	1.82 (1.02,3.26), ***P*=0.043**	1.40 (0.77,2.56), *P*=0.273	1.15 (0.62,2.12), *P*=0.655

^a^Model 1 and ^b^model 2. OR and aOR of AA per doubling in concentration.

Bold values: significant associations.

### 3.2 Aortic diameter and the association with markers of platelet activation, hemostasis, and endothelial disruption

**Figure 2:**
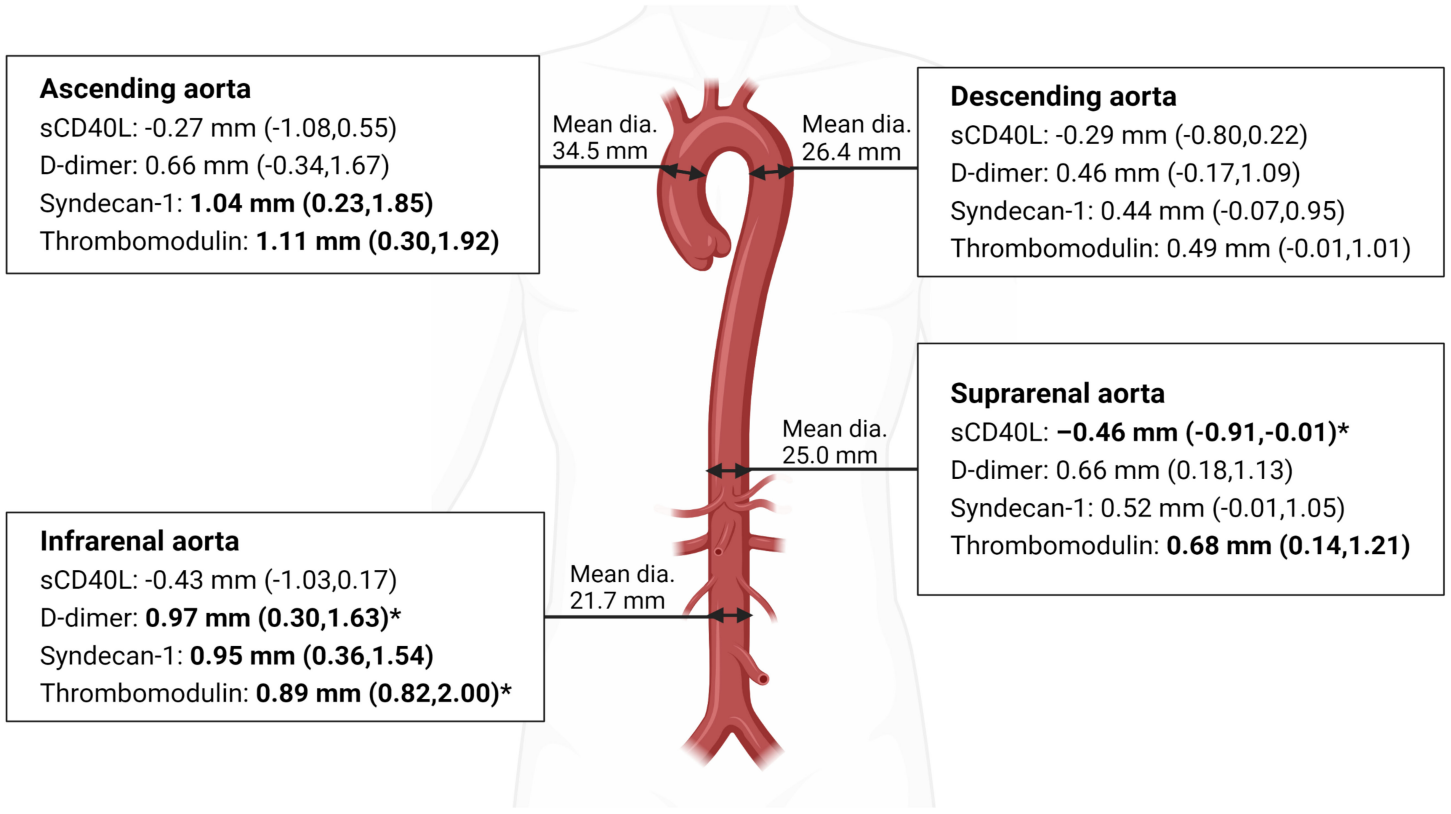
Aortic diameter measured as maximum outer diameter. Mean diameter and larger or smaller diameter in mm (95% CI) associated with high level of the biomarker. Bold text: significant associations. *Adjusted for age, sex, hypertension, smoking status, and BMI category. (Made with biorender.com).

The mean (SD) aortic diameters are presented in **Figure 2** and **Table 4**. Diameter in ascending aorta 34.54 mm (4.30); in descending aorta 26.36 mm (2.70); in suprarenal aorta 24.97 mm (2.82); and in infrarenal aorta 21.65 mm (3.17). In sensitivity analyses, high level of sCD40L was associated with smaller suprarenal aortic diameter (0.46 mm (95% CI 0.01-0.91; *P*=0.045)) adjusted in model 2 but not univariably (0.40 mm (95% CI 0.14-0.93; *P*=0.144)). Detectable level of D-dimer was associated with larger infrarenal aortic diameter; univariably 1.11 mm (95% CI 0.38-1.84; *P*=0.003) and 0.97 mm (95% CI 0.30-1.63; *P*=0.004) adjusted in model 2. High level of syndecan-1 was univariably associated with larger diameter of the ascending aorta (1.04 mm (95% CI 0.23-1.85; *P*=0.012)) and with larger infrarenal aortic diameter (0.95 mm (95% CI 0.36-1.55; *P*=0.002)). High level of thrombomodulin was associated with larger infrarenal aortic diameter; univariably 1.41 mm (95% CI 0.82-2.00; *P*<0.0001) and 0.89 mm (95% CI 0.38-1.41; *P*<0.001) adjusted in model 2. Furthermore, high level of thrombomodulin was univariably associated with larger diameter of the ascending aorta (1.12 mm (95% CI 0.30-1.92; *P*=0.007)) and with larger suprarenal aortic diameter (0.68 mm (95% CI 0.14-1.21; *P*=0.024)) (**Table 4** and **Figure 2**).

**Table 4:** Associations between aortic diameter and the biomarkers.

Variable	Larger or smaller diameter in mm if high level (95% CI), P-value	Larger or smaller diameter in mm if high level^a^ (95% CI), P-value	Larger or smaller diameter in mm if high level^b^ (95% CI), P-value
Ascending aorta			
sCD40L	-0.27 (-1.08,0.55), *P*=0.519	-0.18 (-0.92,0.57), *P*=0.643	-0.47 (-1.20,0.27), *P*=0.215
D-dimer	0.66 (-0.34,1.67), *P*=0.196	0.29 (-0.63,1-21), *P*=0.538	0.33 (-0.62,1.27), *P*=0.494
Syndecan-1	1.04 (0.23,1.85), ***P*=0.012**	0.51 (-0.24,1.26), *P*=0.182	0.54 (-0.22,1.29), *P*=0.167
Thrombomodulin	1.11 (0.30,1.92), ***P*=0.007**	0.68 (-0.06,1.43), *P*=0.073	0.59 (-0.16,1.34), *P*=0.121
Descending aorta			
sCD40L	-0.29 (-0.80,0.22), *P*=0.266	-0.17 (-0.59,0.24), *P*=0.408	-0.37 (-0.76,0.03), *P*=0.069
D-dimer	0.46 (-0.17,1.09), *P*=0.154	0.16 (-0.35,0.67), *P*=0.535	0.21 (-0.29,0.71), *P*=0.415
Syndecan-1	0.44 (-0.07,0.95), *P*=0.094	-0.08 (-0.50,0.34), *P*=0.723	-0.05 (-0.45,0.36), *P*=0.821
Thrombomodulin	0.49 (-0.01,1.01), *P*=0.057	0.12 (-0.29,0.54), *P*=0.573	0.10 (-0.30,0.50), *P*=0.636
Suprarenal aorta			
sCD40L	-0.40 (-0.93,0.14), *P*=0.144	-0.33 (-0.77,0.11), *P*=0.150	-0.46 (-0.91,-0.01), ***P*=0.045**
D-dimer	0.66 (0.18,1.13), *P*=0.056	0.35 (-0.23,0.92), *P*=0.235	0.38 (-0.21,0.97), *P*=0.213
Syndecan-1	0.52 (-0.01,1.05), *P*=0.057	0.04 (-0.41,0.49), *P*=0.860	0.01 (-0.46,0.47), *P*=0.980
Thrombomodulin	0.68 (0.14,1.21), ***P*=0.024**	0.29 (-0.15,0.74), *P*=0.203	0.26 (-0.19,0.72), *P*=0.1259
Infrarenal aorta			
sCD40L	-0.43 (-1.03,0.17), *P*=0.158	-0.32 (-0.85,0.21), *P*=0.239	-0.40 (-0.91,0.11), *P*=0.122
D-dimer	1.11 (0.38,1.84), ***P*=0.003**	0.88 (0.24,1.53), ***P*=0.008**	0.97 (0.30,1.63), ***P*=0.004**
Syndecan-1	0.95 (0.36,1.54), ***P*=0.002**	0.46 (-0.08,0.99), *P*=0.095	0.32 (-0.20,0.84), *P*=0.232
Thrombomodulin	1.41 (0.82,2.00), ***P*<0.0001**	1.06 (0.54,1.59), ***P*<0.0001**	0.89 (0.38,1.41), ***P*<0.001**

^a^Model 1 and ^b^model 2. Larger or smaller diameter in mm associated with high levels of the biomarkers.

Bold values: significant associations.

### 3.3 Aortic wall thickness and the association with markers of platelet activation, hemostasis, and endothelial disruption

The mean (SD) aortic wall thickness was: in ascending aorta 2.28 mm (0.59); in descending aorta 2.32 mm (0.60); in suprarenal aorta 2.22 mm (0.76); and in infrarenal aorta 2.09 mm (0.81). In sensitivity analyses, high level of sCD40L was not associated with aortic wall thickness. Detectable level of D-dimer was associated with greater infrarenal aortic wall thickness; univariably 0.46 mm (95% CI 0.36-0.56; *P*<0.001) and 0.41 mm (95% CI 0.30-0.51; *P*<0.001) adjusted in model 2. High level of syndecan-1 was univariably associated with greater infrarenal aortic wall thickness (0.16 mm (95% CI 0.01-0.31; *P*<0.040)). High level of thrombomodulin was associated with greater infrarenal aortic wall thickness; univariably 0.27 mm (95% CI 0.12-0.42; *P*<0.001) and 0.18 mm (95% CI 0.03-0.33; *P*=0.018) adjusted in model 2.

## 4 Discussion

In a large cohort of well-treated PLWH, we found high level of sCD40L to be associated with lower odds of AA and D-dimer above 290 ng/mL (FEU) to be associated with higher odds of AA. The same associations were found in analyses with biomarkers treated as continuous analyses.

### 4.1 sCD40L

The primary source of sCD40L is activated platelets (33). A study reported decreased platelet counts in HIV-negative AA patients compared to controls (34), and it is possible that lower platelet count in the PLWH with AA may contribute to the inverse association between AA and sCD40L, since platelets are critical for vascular endothelial integrity and health (35). Other studies have reported aspirin to suppress (36) or block (37) the release of sCD40L from platelets. Furthermore, hypertension is associated with higher concentration of sCD40L (26), statins reduce sCD40L in CVD patients (38, 39), and alcohol is proposed to partially activate platelets (40) resulting in decreased sCD40L production. However, our analyses showed no such associations, so this cannot explain the contrast to previous reports of sCD40L being increased in plasma from HIV-negative patients with abdominal AA compared to matched controls (11) and the lacking finding of associations between sCD40L and AA (41).

In line with the emerging focus on the role of endothelial cells in AA pathogenesis (9, 10), we hypothesized that the biomarkers may be involved in the pathogenesis prior to the aortic diameter exceeding the aneurysmal limit. Therefore, we performed sensitivity analyses regarding associations between aortic diameter and the biomarkers. We found sCD40L to be negatively associated with suprarenal aortic diameter, while, at present, studies regarding associations between aortic diameter and sCD40L have not been published. Another measurement of interest is the aortic wall thickness, which studies have suggested the incorporation of into the risk analysis of AA (42, 43). Therefore, we investigated associations between wall thickness and the biomarkers but found no association with sCD40L.

### 4.2 D-dimer

D-dimer, an important marker of hemostasis, has been reported elevated in HIV-negative patients with abdominal AA compared to controls without abdominal AA (13–17). Furthermore, a study reported D-dimer to be 49% higher in PLWH on ART aged 45-76 years than in population controls (adjusted percent difference) (27). In line with these findings, our results highlight D-dimer as a possible biomarker, not only associated with abdominal AA, but all AA in PLWH. However, adjusting for known risk factors resulted in an only borderline significant parameter estimate, perhaps caused by an insufficient sample size, as plasma from 83 patients lacked measurement of D-dimer. The reported association between D-dimer and larger infrarenal aortic diameter is consistent with a previous study (44) and suggests a possible role of D-dimer as an indicator of larger infrarenal aortic diameter. Additionally, the reported associations between D-dimer and greater infrarenal aortic wall thickness may indicate increased fibrinolytic activity in the infrarenal aorta. However, as D-dimer is a general marker of fibrinolysis and not disease-specific, combination with other biomarkers may increase the specificity.

### 4.3 Syndecan-1

A previous study reported concentrations of syndecan-1, a key constituent of the endothelial glycocalyx and a regulator of inflammation, to be elevated in aortic tissue, though not in plasma, from patients with ascending AA compared to matched controls (18). Additionally, a study reported elevated syndecan-1 in PLWH compared to uninfected controls (23). Syndecan-1 is a key constituent of glycocalyx on most endothelial cells in the body, and its soluble form is generated by shedding from the endothelial glycocalyx indicative of damage to endothelial cells (45). As suggested by a previous study (18), it is possible that increased local shedding of syndecan-1 from the aneurysm is masked by the total pool of the biomarker, resulting in non-significant associations with AA in this study. Furthermore, since we only measured syndecan-1 in plasma and not aortic tissue, this may altogether explain why we do not reproduce the previously found association. Syndecan-1 being univariably associated with larger diameter in ascending and infrarenal aorta suggests an association between larger aortic diameter and endothelial disruption.

### 4.4 Thrombomodulin

Another biomarker shed from endothelial cells indicating endothelial cell injury (46), is soluble thrombomodulin, which has been reported elevated in serum from HIV-negative patients with abdominal AA compared to healthy controls (21, 22). Moreover, several studies have reported thrombomodulin to be elevated in PLWH compared to uninfected controls (24, 25). We found thrombomodulin to be univariably associated with AA when kept as a continuous variable, but not when used as a dichotomous variable. However, this finding was not significant when adjusted for known risk factors in our predefined models and we considered it to be an incidental finding. A potential reason why we found no independent association between thrombomodulin and AA in PLWH is the above-mentioned possible masking of shedding from the aneurysm by the shedding from all endothelial cells. The reported association between thrombomodulin and larger infrarenal aortic diameter suggests thrombomodulin as a possible marker of aortic diameter, though at present, no studies regarding this association have been published. Moreover, as with syndecan-1, the association between thrombomodulin and greater infrarenal aortic diameter indicates an association between larger aortic diameter and endothelial disruption. However, further studies into these associations are required.

### 4.5 Diameter of aneurysms

The patients in the majority of the previous studies were undergoing surgery for AA, meaning that the diameters of the aneurysms were larger than those in our study, in which the vast majority of aneurysms had diameters below the surgical threshold. A study reported the size of abdominal AA to be associated with concentration of D-dimer (47), while another study suggested endothelin-1 (ET-1), which is released from endothelial cells in response to among other factors vascular injury, as a marker of aneurysm diameter (48). Though such associations have not presently been reported regarding the other biomarkers, it is possible that the smaller diameters of the aneurysms in our study precluded the use of these biomarker concentrations to identify AA, and may, thus, partly explain the negative inverse association between sCD40L and AA and the lack of associations between AA and high levels of syndecan-1 and thrombomodulin, respectively.

### 4.6 Strengths and limitations

A limitation of this study is its cross-sectional design, making it difficult to investigate causal relationships between the biomarkers and AA. Moreover, the cohort is predominantly male and in fact, all AA were found in men. Strengths include a large cohort of well-treated PLWH and the opportunity to adjust for known risk factors for AA. Lastly, the prevalence of AA is high, making it possible to investigate associations with AA.

## 5 Conclusions

In conclusion, in this large cohort of well-treated PLWH, we found high sCD40L to be associated with lower odds of AA, and we confirmed previous findings of the association between high D-dimer and higher odds of AA. Furthermore, thrombomodulin and D-dimer were associated with larger aortic diameter and sCD40L with smaller aortic diameter. These findings regarding sCD40L and AA call for further investigation into its utilization as a biomarker of AA in PLWH. D-dimer holds promise as a possible marker, though not specific of AA, why further examinations of specific biomarkers remain necessary to aid in the early diagnosis of AA in PLWH.

## Data availability statement

The raw data supporting the conclusions of this article will be made available by the authors, without undue reservation.

## Ethics statement

The studies involving human participants were reviewed and approved by Regional Ethical Committee of Copenhagen. The patients/participants provided their written informed consent to participate in this study.

## Author contributions

Design of the COCOMO study was carried out by SN et.al (29). and data collection was performed by the COCOMO-team from the Viro-Immunology Research Group. SO contributed to the Luminex^®^ analysis and interpretation of the analysed biomarkers. Statistical analysis plan and design of this sub study was written by SG under supervision by SN and JH. Statistical analyses, interpretation of data, and writing of the manuscript was performed by SKG under supervision by SN, JH and AK. Review of the manuscript was performed by MP, PS, AF, JK, LK, JG, TB, SO, and KK. All authors contributed to the article and approved the submitted version.

## References

[1] Smit M Brinkman K Geerlings S Smit C Thyagarajan K van Sighem AV. Future challenges for clinical care of an ageing population infected with HIV: a modelling study. Lancet Infect Dis (2015) 15(7):810–8. doi: 10.1016/S1473-3099(15)00056-0 PMC4528076 26070969

[2] Gebo KA. Epidemiology of HIV and response to antiretroviral therapy in the middle aged and elderly. Aging Health (2008) 4(6):615. doi: 10.2217/1745509X.4.6.615 19915688 PMC2776752

[3] Shah ASV Stelzle D Ken Lee K Beck EJ Alam S Clifford S. Global burden of atherosclerotic cardiovascular disease in people living with HIV: Systematic review and meta-analysis. Circulation (2018) 138(11):1100–12. doi: 10.1161/CIRCULATIONAHA.117.033369 PMC6221183 29967196

[4] Neuhaus J Angus B Kowalska JD La Rosa A Sampson J Wentworth D. Risk of all-cause mortality associated with non-fatal AIDS and serious non-AIDS events among adults infected with HIV. AIDS (2010) 24(5):697. doi: 10.1097/QAD.0b013e3283365356 20177360 PMC2897168

[5] Høgh J Pham MHC Knudsen AD Thudium RF Gelpi M Sigvardsen PE. HIV Infection is associated with thoracic and abdominal aortic aneurysms: a prospective matched cohort study. Eur Heart J (2021) 42(30):2924–31. doi: 10.1093/eurheartj/ehab348 34240121

[6] Kent KC. Clinical practice. abdominal aortic aneurysms. N Engl J Med (2014) 371(22):2101–8. doi: 10.1056/NEJMcp1401430 25427112

[7] Erbel R Aboyans V Boileau C Bossone E Bartolomeo RD Eggebrecht H. 2014 ESC guidelines on the diagnosis and treatment of aortic diseases: Document covering acute and chronic aortic diseases of the thoracic and abdominal aorta of the adult. Eur Heart J (2014) 35(41):2873–926. doi: 10.1093/eurheartj/ehu281 25173340

[8] Mathur A Mohan V Ameta D Gaurav B Haranahalli P. Aortic aneurysm. J Transl Intern Med (2016) 4(1):35–41. doi: 10.1515/jtim-2016-0008 PMC5290913 28191516

[9] Franck G Dai J Fifre A Ngo S Justine C Michineau S. Reestablishment of the endothelial lining by endothelial cell therapy stabilizes experimental abdominal aortic aneurysms. Circulation (2013) 127(18):1877–87. doi: 10.1161/CIRCULATIONAHA.113.001677 23572502

[10] Siasos G Mourouzis K Oikonomou E Tsalamandris S Tsigkou V Vlasis K. The role of endothelial dysfunction in aortic aneurysms. Curr Pharm Des (2015) 21(28):4016–34. doi: 10.2174/1381612821666150826094156 26306838

[11] Touat Z Ollivier V Dai J Huisse MG Bezeaud A Sebbag U. Renewal of mural thrombus releases plasma markers and is involved in aortic abdominal aneurysm evolution. Am J Pathol (2006) 168(3):1022–30. doi: 10.2353/ajpath.2006.050868 PMC1606522 16507915

[12] Kusters PJH Seijkens TTP Beckers L Lievens D Winkels H De Waard V. CD40L deficiency protects against aneurysm formation. Arterioscler Thromb Vasc Biol (2018) 38:1076–85. doi: 10.1161/ATVBAHA.117.310640 29519940

[13] Golledge J Tsao PS Dalman RL Norman PE. Circulating markers of abdominal aortic aneurysm presence and progression. Circulation (2008) 118(23):2382–92. doi: 10.1161/CIRCULATIONAHA.108.802074 PMC2752737 19047592

[14] Sidloff DA Stather PW Choke E Bown MJ Sayers RD. A systematic review and meta-analysis of the association between markers of hemostasis and abdominal aortic aneurysm presence and size. J Vasc Surg (2014) 59(2):528–35. doi: 10.1016/j.jvs.2013.10.088 24461868

[15] Golledge J Muller R Clancy P McCann M Norman PE. Evaluation of the diagnostic and prognostic value of plasma d-dimer for abdominal aortic aneurysm. Eur Heart J (2011) 32(3):354–64. doi: 10.1093/eurheartj/ehq171 20530504

[16] Takagi H Manabe H Kawai N Goto S Umemoto T. Plasma fibrinogen and d-dimer concentrations are associated with the presence of abdominal aortic aneurysm: a systematic review and meta-analysis. Eur J Vasc Endovasc Surg (2009) 38(3):273–7. doi: 10.1016/j.ejvs.2009.05.013 19560946

[17] Vega De Ceniga M Esteban M Barba A Estallo L Blanco-Colio LM Martin-Ventura JL. Assessment of biomarkers and predictive model for short-term prospective abdominal aortic aneurysm growth-a pilot study. Ann Vasc Surg (2014) 28(7):1642–8. doi: 10.1016/j.avsg.2014.02.025 24632318

[18] Ntika S Tracy LM Franco-Cereceda A Björck HM Krizhanovskii C. Syndecan-1 expression is increased in the aortic wall of patients with type 2 diabetes but is unrelated to elevated fasting plasma glucagon-like peptide-1. Biomedicines (2021) 9(6):697. doi: 10.3390/biomedicines9060697 34203009 PMC8233803

[19] Wen J Wang P Smith SV Haller CA Chaikof EL. Syndecans are differentially expressed during the course of aortic aneurysm formation. J Vasc Surg (2007) 46(5):1014–25. doi: 10.1016/j.jvs.2007.06.022 17905554

[20] Xiao J Angsana J Wen J Smith SV Park PW Ford ML. Syndecan-1 displays a protective role in aortic aneurysm formation by modulating T cell-mediated responses. Arterioscler Thromb Vasc Biol (2012) 32(2):386–96. doi: 10.1161/ATVBAHA.111.242198 PMC3404811 22173227

[21] Budzyń M Gryszczyńska B Majewski W Krasiński Z Kasprzak MP Formanowicz D. The association of serum thrombomodulin with endothelial injuring factors in abdominal aortic aneurysm. BioMed Res Int (2017) 2017:2791082. doi: 10.1155/2017/2791082 28473982 PMC5394357

[22] Brunelli T Prisco D Fedi S Rogolino A Farsi A Marcucci R. High prevalence of mild hyperhomocysteinemia in patients with abdominal aortic aneurysm. J Vasc Surg (2000) 32(3):531–6. doi: 10.1067/mva.2000.107563 10957660

[23] Meneses GC Cavalcante MG Da Silva Junior GB Martins AMC Da Justa Pires Neto R Libório AB. Endothelial glycocalyx damage and renal dysfunction in HIV patients receiving combined antiretroviral therapy. AIDS Res Hum Retroviruses (2017) 33(7):703–10. doi: 10.1089/aid.2016.0284 28260391

[24] Leucker TM Weiss RG Schär M Bonanno G Mathews L Jones SR. Coronary endothelial dysfunction is associated with elevated serum PCSK9 levels in people with HIV independent of low-density lipoprotein cholesterol. J Am Heart Assoc (2018) 7(19):e009996. doi: 10.1161/JAHA.118.009996 30371326 PMC6404863

[25] De Larrañaga GF Bocassi AR Puga LM Alonso BS Benetucci JA. Endothelial markers and HIV infection in the era of highly active antiretroviral treatment. Thromb Res (2003) 110(2–3):93–8. doi: 10.1016/S0049-3848(03)00291-3 12893023

[26] Falasca K Reale M Di Nicola M Ucciferri C Zecca IA Santilli F. Circulating CD40 ligand, dickkopf-1 and p-selectin in HIV-infected patients. HIV Med (2019) 20(10):681–90. doi: 10.1111/hiv.12789 31424619

[27] Neuhaus J Jacobs DR Baker JV Calmy A Duprez D La Rosa A. Markers of inflammation, coagulation and renal function are elevated in adults with HIV infection. J Infect Dis (2010) 201(12):1788. doi: 10.1086/652749 20446848 PMC2872049

[28] Haugaard AK Lund TT Birch C Rönsholt F Trøseid M Ullum H. Discrepant coagulation profile in HIV infection: elevated d-dimer but impaired platelet aggregation and clot initiation. AIDS (2013) 27(17):2749–58. doi: 10.1097/01.aids.0000432462.21723.ed 23842126

[29] Ronit A Haissman J Kirkegaard-Klitbo DM Kristensen TS Lebech AM. Benfield T, et al. Copenhagen comorbidity in HIV infection (COCOMO) study: a study protocol for a longitudinal, non-interventional assessment of non-AIDS comorbidity in HIV infection in Denmark. BMC Infect Dis (2016) 16(1):713. doi: 10.1186/s12879-016-2026-9 27887644 10.1186/s12879-016-2026-9 PMC5124288

[30] James PA Oparil S Carter BL Cushman WC Dennison-Himmelfarb C Handler J. 2014 Evidence-based guideline for the management of high blood pressure in adults: report from the panel members appointed to the eighth joint national committee (JNC 8). JAMA (2014) 311(5):507–20. doi: 10.1001/jama.2013.284427 24352797

[31] Lindholt JS Rasmussen LM Søgaard R Lambrechtsen J Steffensen FH Frost L. Baseline findings of the population-based, randomized, multifaceted Danish cardiovascular screening trial (DANCAVAS) of men aged 65-74 years. Br J Surg (2019) 106(7):862–71. doi: 10.1002/bjs.11135 30919411

[32] R Studio Team. R stud. Boston, MA: R Studio Team (2015).

[33] Aloui C Prigent A Sut C Tariket S Hamzeh-Cognasse H Pozzetto B. The signaling role of cd40 ligand in platelet biology and in platelet component transfusion. Int J Mol Sci (2014) 15(12):22342–64. doi: 10.3390/ijms151222342 PMC4284712 25479079

[34] Milne AA Adam DJ Murphy WG Ruckley CV. Effects of asymptomatic abdominal aortic aneurysm on the soluble coagulation system, platelet count and platelet activation. Eur J Vasc Endovasc Surg (1999) 17(5):434–7. doi: 10.1053/ejvs.1998.0790 10329529

[35] Nachman RL Rafii S. Platelets, petechiae, and preservation of the vascular wall. N Engl J Med (2008) 359(12):1261. doi: 10.1056/NEJMra0800887 18799560 PMC2935201

[36] Enomoto Y Adachi S Matsushima-Nishiwaki R Doi T Niwa M Akamatsu S. Thromboxane A(2) promotes soluble CD40 ligand release from human platelets. *Atherosclerosis* . (2010) 209(2):415–21. doi: 10.1016/j.atherosclerosis.2009.10.024 19932480

[37] Nannizzi-Alaimo L Alves VL Phillips DR. Inhibitory effects of glycoprotein IIb/IIIa antagonists and aspirin on the release of soluble CD40 ligand during platelet stimulation. Circulation (2003) 107(8):1123–8. doi: 10.1161/01.CIR.0000053559.46158.AD 12615789

[38] Li J Zhao SP Peng DQ Xu ZM Zhou H. Early effect of pravastatin on serum soluble CD40L, matrix metalloproteinase-9, and c-reactive protein in patients with acute myocardial infarction. Clin Chem (2004) 50(9):1696–9. doi: 10.1373/clinchem.2003.030940 15265816

[39] Schönbeck U Gerdes N Varo N Reynolds RS Horton DB Bavendiek U. Oxidized low-density lipoprotein augments and 3-hydroxy-3-methylglutaryl coenzyme a reductase inhibitors limit CD40 and CD40L expression in human vascular cells. Circulation (2002) 106(23):2888–93. doi: 10.1161/01.CIR.0000043029.52803.7B 12460867

[40] Salem RO Laposata M. Effects of alcohol on hemostasis. Am J Clin Pathol (2005) 123(Suppl 1):S96–105. doi: 10.1309/113N8EUFXYUECCNA 16100871

[41] Flondell-Sité D Lindblad B Kölbel T Gottsäter A. Cytokines and systemic biomarkers are related to the size of abdominal aortic aneurysms. Cytokine (2009) 46:211–5. doi: 10.1016/j.cyto.2009.01.007 19251434

[42] Shang EK Nathan DP Woo EY Fairman RM Wang GJ Gorman RC. Local wall thickness in finite element models improves prediction of abdominal aortic aneurysm growth. J Vasc Surg (2015) 61(1):217–23. doi: 10.1016/j.jvs.2013.08.032 PMC6585429 24095043

[43] Shang EK Nathan DP Sprinkle SR Fairman RM Bavaria JE Gorman RC. Impact of wall thickness and saccular geometry on the computational wall stress of descending thoracic aortic aneurysms. Circulation (2013) 128(11 Suppl 1):S157–62. doi: 10.1161/CIRCULATIONAHA.112.000200 24030401

[44] Laughlin GA Allison MA Jensky NE Aboyans V Wong ND Detrano R. Abdominal aortic diameter and vascular atherosclerosis: the multi-ethnic study of atherosclerosis. Eur J Vasc Endovasc Surg (2011) 41(4):481–7. doi: 10.1016/j.ejvs.2010.12.015 PMC3070051 21236707

[45] Bartlett AH Hayashida K Park PW. Molecular and cellular mechanisms of syndecans in tissue injury and inflammation. Mol Cells (2007) 24(2):153–66. 17978567

[46] Martin FA Murphy RP Cummins PM. Thrombomodulin and the vascular endothelium: insights into functional, regulatory, and therapeutic aspects. Am J Physiol Heart Circ Physiol (2013) 304(12):H1585–97. doi: 10.1152/ajpheart.00096.2013 PMC7212260 23604713

[47] Yamazumi K Ojiro M Okumura H Aikou T. An activated state of blood coagulation and fibrinolysis in patients with abdominal aortic aneurysm. Am J Surg (1998) 175(4):297–301. doi: 10.1016/S0002-9610(98)00014-2 9568655

[48] Třeška V Wenham PW Valenta J Topolčan O Pecen L. Plasma endothelin levels in patients with abdominal aortic aneurysms. Eur J Vasc Endovasc Surg (1999) 17(5):424–8. doi: 10.1053/ejvs.1998.0800 10329527

